# Supplementary material for: Microbiota composition and correlations with environmental factors in grass carp (Ctenopharyngodon idella) culture ponds in South China
Source: PeerJ. 2023 Oct 12;11:e15892. doi: 10.7717/peerj.15892 (PMC10576968; doi:10.7717/peerj.15892)
Supplement: Supplemental Information 1 — Figure S1. Cladogram profiles show the LEfSe results between water and sediment microbiota in different kinds of ponds cultured with different sizes of grass carp. Figure S2. Pearson correlation between physicochemical indices and microbiota in water (A) and sediment (B) of different types of ponds cultured with different sizes of grass carp. * p < 0.05; ** p < 0.01; *** p < 0.001. Table S1. Differences of Richness, Shannon, and ACE indices of water and sediment microbiota between different ponds with the same type. Four types of ponds with 3 ponds for each type were investigated. Three surface waters approximately 50 cm below the water level (approximately 1 L) and three upper (0–8 cm) sediment (approximately 500 g) samples were collected from the left, center, and right of each pond using a 5 L hydrophore sampler and Van Veen Grab sampler to study, respectively. LF, larval fish; SJ, small juvenile fish; MJ, middle juvenile fish; LJ, large juvenile fish. [file peerj-11-15892-s001.docx]

Figure S1. Cladogram profiles show the LEfSe results between water and sediment microbiota in different kinds of ponds cultured with different sizes of grass carp.


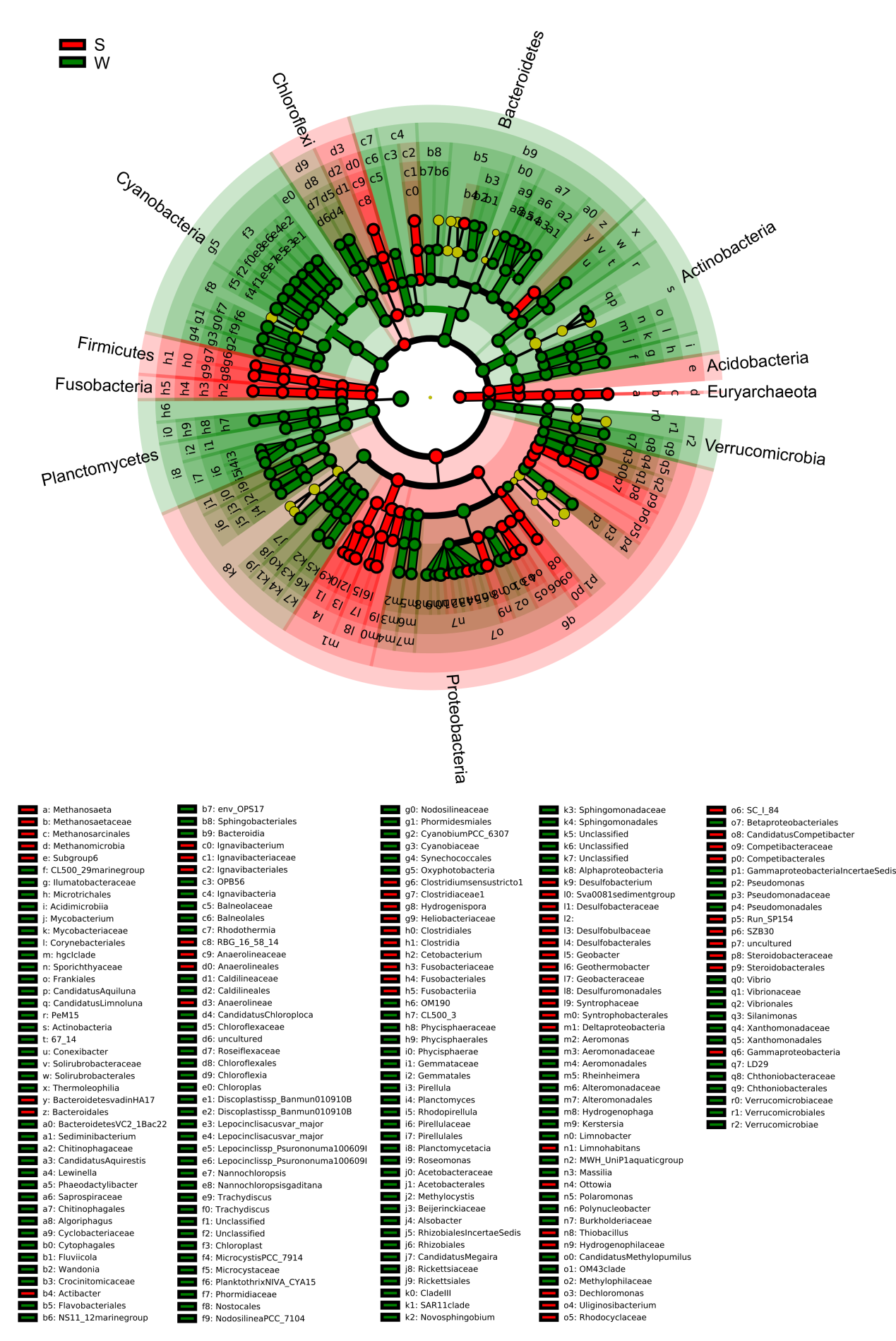


Figure S2. Pearson correlation between physicochemical indices and microbiota in water (A) and sediment (B) of different types of ponds cultured with different sizes of grass carp. * p < 0.05; ** p < 0.01; *** p < 0.001.


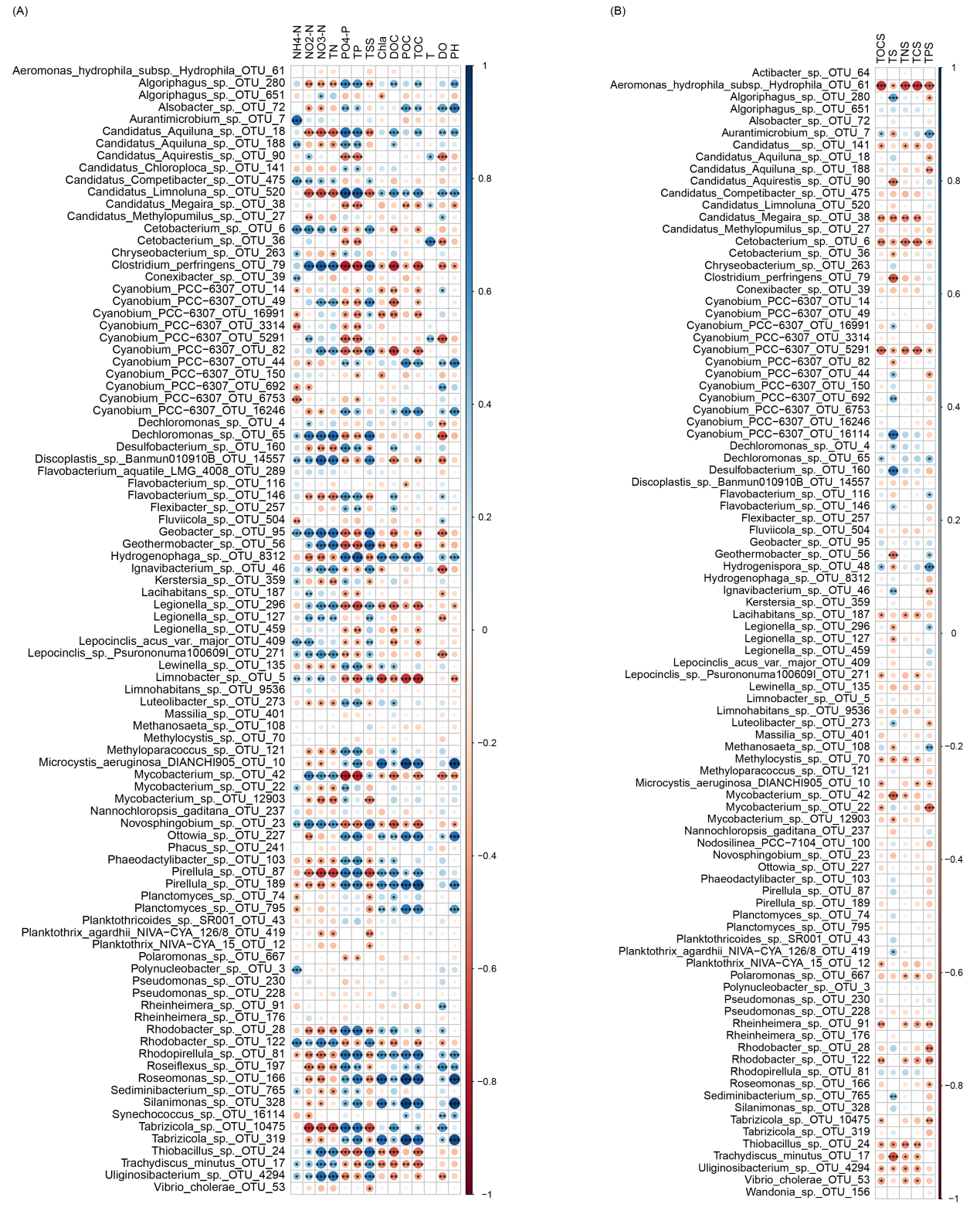


Table S1. Differences of Richness, Shannon, and ACE indices of water and sediment microbiota between different ponds with the same type. Four types of ponds with 3 ponds for each type were investigated. Three surface waters approximately 50 cm below the water level (approximately 1 L) and three upper (0-8 cm) sediment (approximately 500 g) samples were collected from the left, center, and right of each pond using a 5 L hydrophore sampler and Van Veen Grab sampler to study, respectively. LF, larval fish; SJ, small juvenile fish; MJ, middle juvenile fish; LJ, large juvenile fish.

| Habitat | Type of pond | Pond | Shannon index | Richness | ACE index |
| --- | --- | --- | --- | --- | --- |
| Water | LF | P1 | 4.938±0.347 | 1103.667±318.623 | 1890.142±529.731 |
|  |  | P2 | 5.063±0.178 | 1387.667±302.093 | 2376.877±517.290 |
|  |  | P3 | 4.771±0.307 | 1003±153.522 | 1708.492±267.811 |
|  | *p* | | 0.393 | 0.27 | 0.288 |
|  | SJ | P1 | 5.014±0.152 | 1272.667±25.384 | 1920.060±104.560 |
|  |  | P2 | 5.785±0.161 | 1965.333±359.875 | 3100.249±665.055 |
|  |  | P3 | 5.939±0.094 | 2121.333±292.966 | 3236.874±578.232 |
|  | *p* | | 0.051 | 0.051 | 0.061 |
|  | MJ | P1 | 5.740±0.117 | 2421.667±191.377 | 3913.592±340.939 |
|  |  | P2 | 5.780±0.044 | 2374.333±171.074 | 3758.007±315.455 |
|  |  | P3 | 5.7520±0.069 | 2155±89.202 | 3544.401±128.272 |
|  | *p* | | 0.875 | 0.113 | 0.393 |
|  | LJ | P1 | 5.896±0.042^b^ | 2629±49.153 | 4147.832±40.666 |
|  |  | P2 | 6.124±0.0549^a^ | 2804.333±127.845 | 4442.563±267.026 |
|  |  | P3 | 6.054±0.0475^ab^ | 2679.333±83.942 | 4335.065±84.640 |
|  | *p* | | 0.039 | 0.148 | 0.193 |
| Sediment | LF | P1 | 6.329±0.163 | 4345.333±70.586 | 6648.171±191.147 |
|  |  | P2 | 6.303±0.225 | 4478.667±246.833 | 6859.606±341.695 |
|  |  | P3 | 6.402±0.213 | 3945±283.175 | 5705.278±653.867 |
|  | *p* | | 0.393 | 0.061 | 0.061 |
|  | SJ | P1 | 6.693±0.173 | 4602.667±150.324^ab^ | 6851.239±29.718^ab^ |
|  |  | P2 | 6.634±0.150 | 4775.333±73.501^a^ | 7184.503±167.462^a^ |
|  |  | P3 | 6.005±0.143 | 4131.333±157.684^b^ | 6344.932±180.330^b^ |
|  | *p* | | 0.066 | 0.039 | 0.027 |
|  | MJ | P1 | 6.951±0.123 | 4888.333±296.092 | 7023.241±511.092 |
|  |  | P2 | 6.881±0.023 | 4787.667±134.931 | 6981.607±113.536 |
|  |  | P3 | 6.994±0.097 | 5265.333±312.182 | 7782.698±504.585 |
|  | *p* | | 0.252 | 0.113 | 0.118 |
|  | LJ | P1 | 6.782±0.012 | 4650±52.115 | 6833.453±208.593 |
|  |  | P2 | 6.813±0.230 | 4861.667±387.500 | 7211.371±558.871 |
|  |  | P3 | 6.782±0.028 | 4738±6.928 | 6887.262±54.172 |
|  | *p* | | 0.733 | 0.298 | 0.67 |

Table S2. Dominant OTUs detected in the water and sediment microbiota in ponds cultured with different sizes of grass carp. Four types of ponds with 3 ponds for each type were investigated. Three surface waters approximately 50 cm below the water level (approximately 1 L) and three upper (0-8 cm) sediment (approximately 500 g) samples were collected from the left, center, and right of each pond using a 5 L hydrophore sampler and Van Veen Grab sampler to study, respectively. LF, larval fish; SJ, small juvenile fish; MJ, middle juvenile fish; LJ, large juvenile fish.

Please see the Excel table for details.
